# Assessment and Management of Anti-Insulin Autoantibodies in Varying Presentations of Insulin Autoimmune Syndrome

**DOI:** 10.1210/jc.2018-00972

**Published:** 2018-07-31

**Authors:** David Church, Luís Cardoso, Richard G Kay, Claire L Williams, Bernard Freudenthal, Catriona Clarke, Julie Harris, Myuri Moorthy, Efthmia Karra, Fiona M Gribble, Frank Reimann, Keith Burling, Alistair J K Williams, Alia Munir, T Hugh Jones, Dagmar Führer, Lars C Moeller, Mark Cohen, Bernard Khoo, David Halsall, Robert K Semple

**Affiliations:** 1 University of Cambridge Metabolic Research Laboratories, Wellcome Trust–MRC Institute of Metabolic Science, Cambridge, United Kingdom; 2 National Institute for Health Research Cambridge Biomedical Research Centre, Cambridge, United Kingdom; 3 Department of Clinical Biochemistry and Immunology, Cambridge University Hospitals, NHS Foundation Trust, Cambridge, United Kingdom; 4 Department of Endocrinology, Diabetes and Metabolism, Centro Hospitalar e Universitário de Coimbra, Coimbra, Portugal; 5 Academic Endocrine Unity, Faculty of Medicine, University of Coimbra, Coimbra, Portugal; 6 Diabetes & Metabolism, Translational Health Sciences, University of Bristol, Southmead Hospital, Bristol, United Kingdom; 7 Department of Diabetes & Endocrinology, Royal Free Hospital, London, NHS Foundation Trust, London, United Kingdom; 8 Department of Clinical Biochemistry, Western General Hospital, NHS Lothian, Edinburgh, United Kingdom; 9 Core Biochemical Assay Laboratory, Cambridge University Hospitals, NHS Foundation Trust, Cambridge, United Kingdom; 10 Department of Endocrinology, Royal Hallamshire Hospital, Sheffield, United Kingdom; 11 Robert Hague Centre for Diabetes and Endocrinology, Barnsley Hospital, NHS Foundation Trust, Barnsley, United Kingdom; 12 Department of Endocrinology, Diabetes and Metabolism, University Hospital Essen, University of Duisburg-Essen, Essen, Germany; 13 University of Edinburgh Centre for Cardiovascular Science, Queen’s Medical Research Institute, Edinburgh, United Kingdom

## Abstract

**Context:**

Insulin autoimmune syndrome (IAS), spontaneous hyperinsulinemic hypoglycemia due to insulin-binding autoantibodies, may be difficult to distinguish from tumoral or other forms of hyperinsulinemic hypoglycemia, including surreptitious insulin administration. No standardized treatment regimen exists.

**Objectives:**

To evaluate an analytic approach to IAS and responses to different treatments.

**Design and Setting:**

Observational study in the UK Severe Insulin Resistance Service.

**Patients:**

Six patients with hyperinsulinemic hypoglycemia and detectable circulating anti–insulin antibody (IA).

**Main Outcome Measures:**

Glycemia, plasma insulin, and C-peptide concentrations by immunoassay or mass spectrometry (MS). Immunoreactive insulin was determined in the context of polyethylene glycol (PEG) precipitation and gel filtration chromatography (GFC). IA quantification using ELISA and RIA, and IA were further characterized using radioligand binding studies.

**Results:**

All patients were diagnosed with IAS (five IgG, one IgA) based on a high insulin/C-peptide ratio, low insulin recovery after PEG precipitation, and GFC evidence of antibody-bound insulin. Neither ELISA nor RIA result proved diagnostic for every case. MS provided a more robust quantification of insulin in the context of IA. One patient was managed conservatively, four were treated with diazoxide without sustained benefit, and four were treated with immunosuppression with highly variable responses. IA affinity did not appear to influence presentation or prognosis.

**Conclusions:**

IAS should be considered in patients with hyperinsulinemic hypoglycemia and a high insulin/C-peptide ratio. Low insulin recovery on PEG precipitation supports the presence of insulin-binding antibodies, with GFC providing definitive confirmation. Immunomodulatory therapy should be customized according to individual needs and clinical response.

## Teaser

A case series of insulin autoimmune syndrome highlighting a range of dysglycemic presentations, diagnostic challenges, and variable responses to therapy.

Insulin autoimmune syndrome (IAS) features hyperinsulinemic hypoglycemia due to insulin autoantibodies in exogenous insulin-naive individuals (1, 2). IAS presents with recurrent postabsorptive or fasting hypoglycemia, alternating with postprandial hyperglycemia, due to “buffering” by autoantibodies, which sequester insulin in immune complexes during the acute phase of insulin secretion, only to release it slowly later, at physiologically inappropriate times.

IAS cannot easily be distinguished on clinical grounds from tumoral or other forms of hyperinsulinemic hypoglycemia, which includes hypoglycemia caused by surreptitious insulin administration (3). Altered kinetics of insulin clearance in the presence of antibody binding also commonly skews insulin/C-peptide molar ratios upward, sometimes dramatically so, as insulin clearance is delayed while C-peptide clearance is unaffected. As insulin/C-peptide molar ratios are often used to discriminate exogenous from endogenous hyperinsulinemic hypoglycemia (4), this raises the risk that maleficent insulin use may be erroneously diagnosed, with potentially decisive implications for criminal and child custody proceedings.

Anti–insulin antibody (IA) assays are not standardized and yield variable, qualitative, or semiquantitative results (5); moreover, detection of IA does not prove the presence of circulating insulin-antibody complexes (6). Methods currently used to confirm hormone-antibody complexes include precipitation with polyethylene glycol (PEG), which is not specific (7), and gel filtration chromatography (GFC), which may be used in conjunction with *ex vivo* addition of insulin to enhance sensitivity (6). Mass spectrometry (MS) methods now offer quantification of insulin (8) that is more robust in the face of antihormone antibody interference than immunoassay (9).

Effective use of different immunosuppressive regimens in IAS has been described, including prednisolone (10), hydrocortisone (11), azathioprine (12), cyclophosphamide (13), mycophenolate mofetil (MMF) (14, 15), rituximab (16), and plasmapheresis (17, 18), but no consensus exists about optimal therapy. We now extend experience by presenting clinical and biochemical characteristics of six patients with varying presentations of IAS and responses to immunosuppression.

## Materials and Methods

### Patients and blood sampling

Studies were performed in accordance with the Declaration of Helsinki (2000). Six exogenous insulin-naive patients presenting with hyperinsulinemic hypoglycemia and a high insulin/C-peptide ratio were evaluated by the UK Severe Insulin Resistance Supraregional Assay Service, Cambridge University Hospitals NHS Foundation Trust, Cambridge.

### Immunoassays and insulin immunocomplex detection

Blood was collected on ice and plasma/serum rapidly separated and frozen at −80°C. Plasma insulin and C-peptide were measured using immunoassay platforms approved for clinical use. PEG precipitation studies were performed as previously published (6), with analyte recovery taken to be the PEG supernatant insulin concentration expressed as a percentage of insulin measured in matched saline-diluted samples. GFC was performed as previously described (6).

Anti-insulin IgG was determined using an in-house human insulin–specific ImmunoCAP ELISA. IA was also determined using a competitive IA RIA (19). In brief, 5 μL serum, neat or diluted with IA-negative serum, was incubated with A14-^125^I-labeled human insulin ± unlabeled synthetic human insulin at 40 μmol/L. ^125^I-IA complexes were precipitated using glycine-blocked protein A Sepharose (PAS), ethanolamine-blocked protein G Sepharose (PGS) (20), and/or IgA agarose.

IA affinity was assessed in neat and diluted serum (21, 22), with immune complexes precipitated using a 50:50 mixture of PAS and PGS to include all possible IA-reactive IgG antibodies. IC50 and Kd (mol/L) were calculated by nonlinear regression analysis using a one-site model (22) (*R*^2^ values of 0.88 to 0.99), assuming equal antibody binding by labeled and unlabeled insulin.

### Immunosubtraction using anti–human immunoglobulin-agarose

Synthetic human insulin, diluted in 5% BSA, was added to plasma before 24-hour incubation at 24°C. Agarose-conjugated anti-immunoglobulin (anti–human IgA, anti–human IgM, and anti–human IgG) was washed thrice with 0.9% saline and stored at 4°C. Agarose conjugates were added to plasma at ratios based on in-house data (volume ratios of agarose-antibody/plasma were 5:1 for anti-IgA, 29:20 for anti-IgM, and 32:3 for anti-IgG). IgA antibody-agarose experiments for patient 6 were performed in triplicate. Samples were mixed for 60 minutes prior to centrifugation at 13,200*g* for 15 minutes. To overcome sampling error due to increased sample viscosity, agarose supernatant was diluted in saline prior to analysis. Insulin recovery was calculated as percentage insulin recovery in agarose supernatant of dilution-matched plasma.

### Quantitative mass-spectrometric analysis of insulin and C-peptide

Pooled human plasma was fortified with insulin lispro and C-peptide to generate concentrations of 8610 pmol/L to 17 pmol/L and 16,548 pmol/L to 33 pmol/L, respectively. Then, 250 μL of each sample of known peptide concentration, available patient plasma, and unfortified pooled plasma was transferred to different wells of a 2-mL 96-well plate. Five patient and 34 control samples were extracted using a combination of acetonitrile precipitation and solid-phase extraction–liquid chromatography (23) along with quality control samples and analyzed with two separately extracted sets of calibration samples. MS data were acquired from *m/z* 700 to 1600, with a resolution of 70,000 and an automatic gain control target of 3e6 ions. Insulin and C-peptide calibration curves were generated using *m/z* values for the [M+5H]^5+^ charge states relating to the monoisotopic (1161.7362) and multiple ^13^C isotopes of human insulin and for the [M+3H]^3+^ charge state of C-peptide (1007.1783). Calibration curves for insulin and C-peptide gave a linear fit with *R*^2^ values of 0.995 and 0.994, respectively, after correcting for endogenous analyte, and calibration standards and quality control samples were all within ±25% of expected values. Regression between immunoassay and MS control plasma values was linear for insulin (0.8727x − 27.025; *R*^2^ = 0.974) and C-peptide (1.317x − 56.86; *R*^2^ = 0.997).

## Results

A summary of the clinical characteristics of patients studied and the investigations undertaken on initial presentation is given in Table 1. Case histories follow.

**Table 1.** Clinical Characteristics and Initial Investigation of Patients Studied

Patient	Age, y	Sex	Ethnicity	BMI, kg/m^2^	Preexisting Diagnoses	Medications	Presentation	Investigations With Abnormal Results ^*a*^	Investigations With Normal Results ^*a*^	Negative Imaging
1	56	Female	Caucasian	26.2	Autoimmune hypothyroidism	None	Postprandial hypoglycemia	OGTT nadir 39 mg/dL (2.2 mmol/L)	HbA1c	CT abdomen
					Asthma			CGMS	72-h fast nadir 59 mg/dL (3.3 mmol/L)	MRI abdomen
					Factor XI deficiency				MMTT	Endoscopic US
									SU screen	
									*α*-Islet, *α*-GAD65, *α*-IA2, *α*-INSR autoantibodies	
2	52	Female	Thai	35.0	None	None	Fasting hypoglycemia		SU screen *α*-INSR autoantibodies	^68^Ga-DOTATATE PET/CT
3	28	Female	Caucasian	25.1	None	None	Fasting hypoglycemia		HbA1c SU screen	^68^Ga-DOTATATE PET/CT
4	76	Male	Caucasian	29.5	Type 2 diabetes, ischemic heart disease, parotid pleomorphic adenoma, glaucoma	Spironolactone, furosemide, losartan, aspirin, bisoprolol, atorvastatin, omeprazole, fluoxetine	Postprandial/ nocturnal hypoglycemia	MMTT nadir 29 mg/dL (1.6 mmol/L)	72-h fast nadir 45 mg/dL (2.5 mmol/L)	MRI abdomen
								CGMS		Endoscopic US
										Octreotide SPECT
										^18^F-Deoxyglucose-PET
5	89	Female	Caucasian	19.4	Small B-cell lymphoma	Furosemide, fexofenadine, ferrous fumarate	Low-capillary blood glucose readings		Short Synacthen test	nil
6	50	Male	Caucasian	22.3	None	None	Postprandial hypoglycemia	OGTT nadir 26 mg/dL (1.4 mmol/L)	72-h fast nadir 72 mg/dL (4.0 mmol/L)	CT abdomen
									SU screen	

Abbreviations: BMI, body mass index; GAD, glutamic acid decarboxylase; IA2, islet antigen-2; MMTT, mixed meal tolerance test; OGTT, oral glucose tolerance test; PET, positron emission tomography; SPECT, single-photon emission computerized tomography; SU, sulfonylurea; US, ultrasound; *α*-INSR, anti–insulin receptor.

^a^ Hypoglycemia with inappropriately elevated plasma insulin was an inclusion criterion for this study and was excluded from the table.

**Figure 1.**
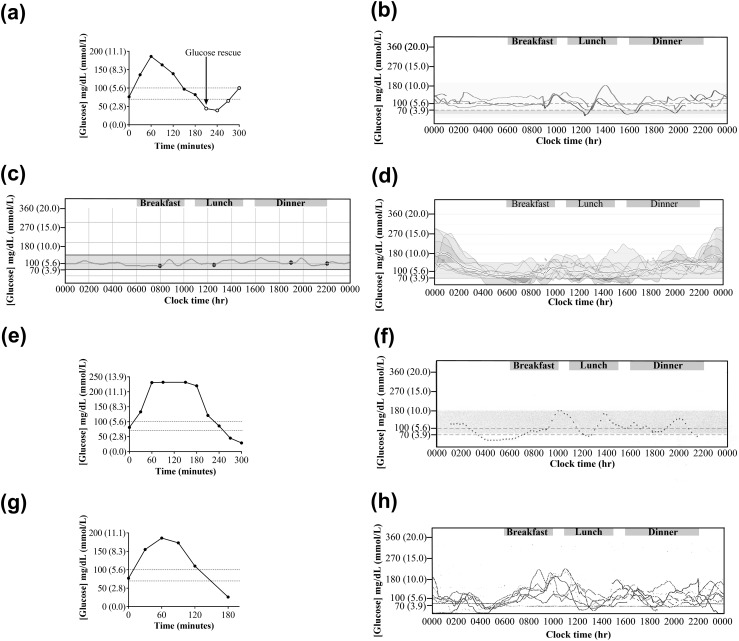
Variable patterns of dysglycemia of patients studied. (a) Venous plasma glucose concentrations during a 75-g OGTT at presentation of patient 1; ○ denotes glucose measurements following glucose rescue. The glucose nadir was 39 mg/dL (2.2 mmol/L). (b) Demonstration of labile glycemia in patient 1 at presentation by CGMS. (c) Demonstration of normoglycemia in patient 1 following immunomodulation therapy. (d) Demonstration of labile glycemia in patient 3 concomitant with glucocorticoid therapy. (e) Demonstration of reactive hypoglycemia in patient 4 at presentation by mixed-meal tolerance test. The peak glucose concentration was 232 mg/dL (12.9 mmol/L) with glucose nadir at 300 minutes of 29 mg/dL (1.6 mmol/L). (f) Demonstration of reactive and nocturnal hypoglycemia in patient 4 at presentation by CGMS. (g) Demonstration of reactive hypoglycemia in patient 6 at presentation by 75-g OGTT. The glucose nadir was 26 mg/dL (1.4 mmol/L). (h) Demonstration of labile glycemia in patient 6 at presentation by CGMS.

Patient 1 presented after 20 months of shakiness, sweating, pallor, and confusion, generally 1 to 2 hours postprandially, which were alleviated by carbohydrate ingestion. She had concurrently gained 7 kg in weight. On emergency admission, plasma glucose concentration was 30 mg/dL (1.7 mmol/L) [normal range (NR), 72 to 110 mg/dL], with concomitantly inappropriate plasma immunoassay insulin and C-peptide concentrations of 267 pmol/L (NR <60) and 899 pmol/L (NR 174 to 960), respectively, and a molar ratio of insulin/C-peptide of 0.30 (NR 0.03 to 0.25) (24, 25). A 72-hour fast and mixed-meal tolerance test failed to solicit hypoglycemia, but a 75-g oral glucose tolerance test (OGTT) produced a glucose nadir of 39 mg/dL (2.2 mmol/L) [Fig. 1(a)] at 240 minutes. A continuous glucose monitoring system (CGMS) demonstrated labile glycemia, including late postprandial hypoglycemia [Fig. 1(b)]. IAs were grossly elevated at 722.4 U/mL (NR <0.4) (RiaRSR IAA, Cardiff, UK).

**Figure 2.**
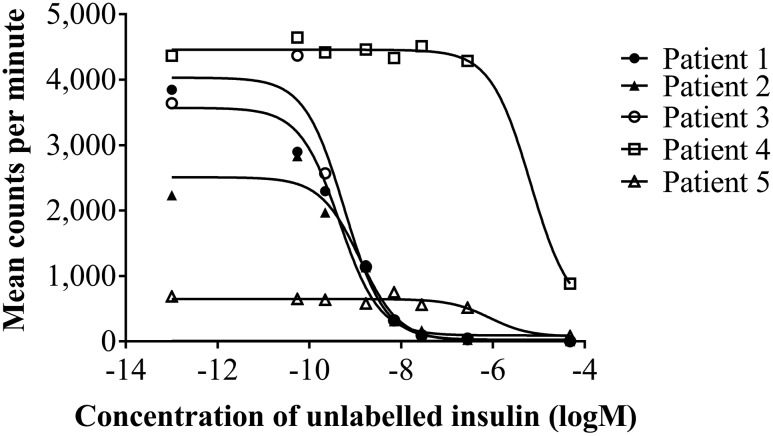
Displacement curves for serum samples from patients 1 to 5 at various dilutions in antibody-negative serum following competitive displacement with unlabeled human insulin. Although identified as low affinity (4.1 × 10^−7^ mol/L), patient 6 plasma was considered unreliable because baseline levels of insulin binding were very low. Serum was diluted as follows: patient 1, 10-fold; patient 2, 50-fold; patient 3, 100-fold; patient 4, neat; patient 5, 10-fold.

Gross hyperinsulinemia was confirmed using MS (Table 2). Low insulin recovery following PEG precipitation using an immunoassay suggested IA. GFC with and without addition of exogenous human insulin showed predominantly high molecular weight (HMW) insulin immunoreactivity, confirming IAS (6). IAs were positive by ELISA and RIA, the latter indicating a high insulin-binding capacity. Competitive insulin-binding studies (Fig. 2) suggested a subnanomolar dissociation constant (analyzed at 10-fold serum dilution, with a two-site model offering the best fit, with both sites binding with high affinity).

**Table 2.** Biochemical Evaluation of Nonfasting Plasma in a Single Specialized Center

Patient No.	MS Insulin, pmol/L	Immunoassay Insulin, pmol/L (<60)		Insulin Recovery After PEG Precipitation, % (>102)	GFC of Insulin	Anti-Insulin IgG, mg/L (0–5) ^*a*^	IA, cIA Units (<0.2)	Kd, mol/L	MS C-peptide, pmol/L	Immunoassay C-peptide, pmol/L (174–960)	MS Insulin/C-peptide Molar Ratio (0.2–1.5)	Immunoassay Insulin/C-Peptide Molar Ratio (0.03–0.25)
		Dilution Ratio (Plasma/Diluent)										
		1:0	1:4									
1	5278	>3000	7020	8	HMW insulin present	16	2408	3.42 × 10^−10^	1428	3750	3.7	1.9
2	—	>3000	11,585	6	HMW insulin present	38	8738	1.16 × 10^−9^	—	5580	—	2.1
3	1583	782	4601	9	HMW insulin present	11	>10,000	4.68 × 10^−10^	215	2380	7.4	0.3
4	2912	1340	3912	11	HMW insulin present	>200	4.0	6.55 × 10^−6^	348	1190	8.4	1.1
5	6589	2781	7805	3	HMW insulin present	89	300	8.55 × 10^−7^	880	3110	7.5	0.9
6	4012	2906	5630	65	HMW insulin present	5	0.1	—	750	3280	5.4	0.9

Abbreviations: cIA, competitive insulin antibody; Kd, dissociation constant.

^a^ The reference range used for the anti-insulin IgG assay was provided by a reference laboratory using the same method (Sheffield Protein Reference Unit, Sheffield, UK). Testing 28 of the 34 control samples used in the quantitative mass-spectrometric analysis of insulin and C-peptide yielded a 75th percentile insulin antibody concentration of 4.8 mg/L.

Two 1-g intravenous methylprednisolone doses were given 1 day apart monthly for 4 months, but symptoms continued over the ensuing 2 years, with hypoglycemia remaining demonstrable on OGTT and CGMS. Rituximab (750 mg/m^2^ × 2) was administered, reducing glycemic lability [Fig. 1(c)], with only two capillary blood glucose (CBG) readings <55 mg/dL (<3.1 mmol/L) recorded over 9 months following rituximab. At this stage, IA concentration had decreased to 153 U/mL (NR <0.4, RiaRSR IAA), and fasting plasma insulin and C-peptide concentrations by immunoassay were 173 pmol/L (NR <60) and 500 pmol/L (NR 174 to 960), respectively.

**Figure 3.**
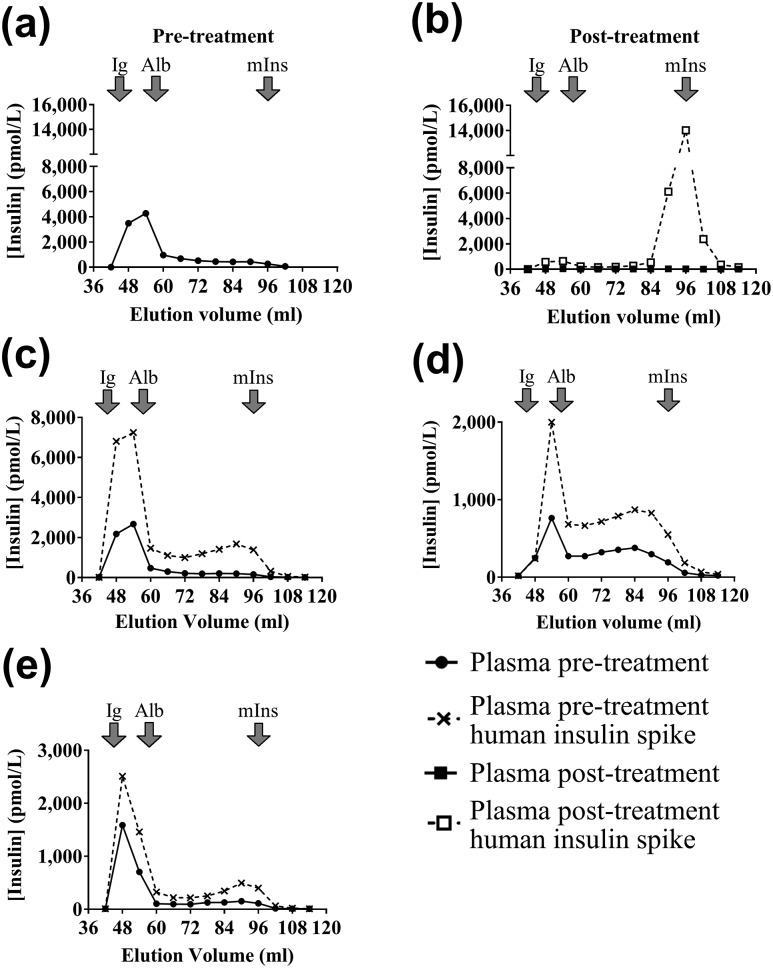
Demonstration of insulin-antibody complexes using GFC. Results of insulin assay after GFC of nonfasting plasma. Elution volumes of immunoglobulin (Ig), albumin (Alb), and monomeric insulin (mIns) are shown. Results are shown for patient 2 at (a) presentation (pretherapy) and (b) with and without preincubation of plasma with exogenous insulin posttherapy, as well as with and without preincubation of plasma with exogenous insulin at presentation for (c) patient 3, (d) patient 4, and (e) patient 6.

Patient 2 presented with fasting symptoms of hypoglycemia, including syncope. She became hypoglycemic after 10 hours of fasting with a venous plasma glucose of 34 mg/dL (1.9 mmol/L) and concomitant plasma insulin immunoassay concentration of 68,123 pmol/L, C-peptide of 3690 pmol/L, and insulin/C-peptide molar ratio of 18 (NR 0.03 to 0.25). Gross hyperinsulinemia was confirmed by immunoassay (Table 2), and low insulin recovery following PEG precipitation suggested IA. GFC of plasma showed HMW insulin immunoreactivity consistent with insulin-binding antibodies, confirming IAS [Fig. 3(a)]. IAs were positive by ELISA and RIA, the latter result consistent with GFC findings of a very high insulin-binding capacity. Competitive insulin-binding studies (Fig. 2) suggested a nanomolar dissociation constant (analyzed at 10- and 50-fold dilution).

Initial diazoxide treatment was ineffective and caused neutropenia, leading to discontinuation. Prednisolone 30 mg daily was begun with addition of MMF 1.5 g daily after IAS confirmation. Hypoglycemia resolved over the subsequent 4 weeks, with anti-insulin IgG falling to 5 mg/L, plasma insulin to 322 pmol/L, and C-peptide to 1210 pmol/L, although insulin recovery after PEG precipitation increased only modestly to 17%. Following treatment, GFC demonstrated a reduction of HMW insulin [Fig. 3(b)]. The patient remained euglycemic on maintenance MMF for 12 months before discontinuing immunosuppressive therapy with no evidence of recurrence during the 12 months of follow-up to date.

Patient 3 presented with 2 years of recurrent anxiety, confusion, perioral paraesthesias, and generalized diaphoresis on fasting. Typically, she would wake during the night with feelings of terror and agitation. These symptoms would swiftly resolve following carbohydrate ingestion. Emergency medical attendants had recorded CBG readings of 36 and 43 mg/dL (2.0 and 2.4 mmol/L). During inpatient supervised fasting, symptomatic hypoglycemia was recorded at 4 hours with a venous plasma glucose of 39 mg/dL (2.2 mmol/L) and paired immunoassay plasma insulin and C-peptide concentrations of 17,800 and 409 pmol/L, respectively, with an insulin to C-peptide molar ratio of 44 (NR 0.03 to 0.25).

Hyperinsulinemia was confirmed using MS (Table 2). Insulin measurement by immunoassay underestimated total insulin in neat plasma and was nonlinear to dilution, with low insulin recovery following PEG precipitation, all suggesting IA. GFC showed predominantly HMW insulin immunoreactivity, confirming the diagnosis of IAS [Fig. 3(c)]. IAs were positive by ELISA and RIA, the latter result consistent with GFC findings of a very high insulin-binding capacity. Competitive insulin-binding studies (Fig. 2) suggested a subnanomolar dissociation constant (analyzed at hundred-fold serum dilution).

**Figure 4.**
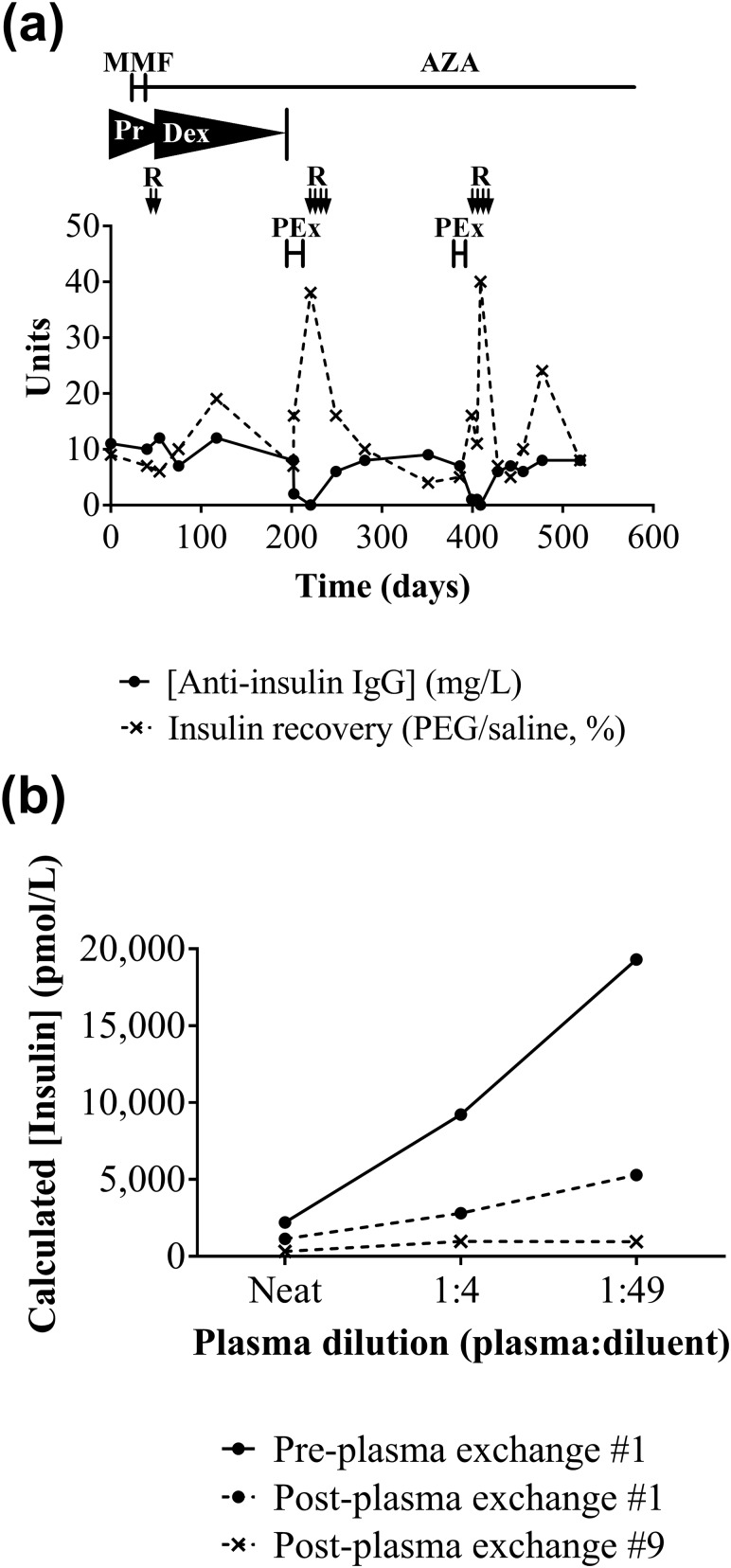
Response of biochemical markers to therapy in patient 3. (a) Cumulative results for patient 3 over course of treatments, including MMF, azathioprine (AZA), prednisolone (Pr), dexamethasone (Dex), rituximab (R), and plasma exchange (PEx), showing anti-insulin IgG concentrations (in-house human insulin–specific ImmunoCAP) and immunoassay insulin recovery following PEG precipitation over time. (b) Effect of plasma exchange on insulin immunoassay linearity to dilution. Calculated insulin concentration plotted against plasma dilution of patient 3 plasma before plasma exchange and following cycle 1 and cycle 9.

Initial diazoxide treatment was ineffective and was discontinued. Prednisolone 60 mg daily, later changed to dexamethasone 8 mg twice daily, was commenced after IAS confirmation, with MMF twice daily later added. CGMS demonstrated both hyperglycemia and hypoglycemia [Fig. 1(d)]. Following nausea and raised serum transaminases, MMF was replaced by azathioprine 50 mg twice daily. High-dose steroid treatment of hypoglycemia produced Cushing syndrome, including agitated depression and avascular necrosis of the hip. Rituximab (1 g × 2) was administered and dexamethasone weaned to 1 mg daily, but no evidence of depletion of the pathogenic antibody [Fig. 4(a)] or glycemic improvement was seen. Plasma exchange (thrice weekly × 8), in contrast, led to resolution of hypoglycemia, disappearance of serum IA, improvement in insulin immunoassay linearity [Fig. 4(b)], and an increase in insulin recovery after PEG precipitation. Although transient, this proved the efficacy of immunodepletion, and plasma exchange followed by a course of rituximab (750 mg/m^2^ × 4) was administered. Despite amelioration of hypoglycemia, euglycemia was not achieved, leading to further plasma exchange and administration of rituximab (750 mg/m^2^ × 4), for recrudescent hypoglycemia 6 months later. After a further 6 months, the patient was taking azathioprine but no glucocorticoid. She no longer had fasting hypoglycemia but had persistent reactive hypoglycemia, managed with dietetic support in combination with acarbose (*α*-glucosidase inhibitor) to limit postprandial insulin secretion.

Patient 4 presented with 9 months of episodic diaphoresis, headache, hunger, and confusion, attributed to spontaneous hypoglycemia. Three days after initial consultation, he had a myocardial infarction and coronary artery bypass surgery. Initial plasma immunoassay insulin concentration was 1732 pmol/L, and C-peptide was 794 pmol/L during spontaneous hypoglycemia. Over two 72-hour fasts, a blood glucose nadir of 45 mg/dL (2.5 mmol/L) was recorded. Mixed-meal tolerance test revealed early postchallenge hyperglycemia, with a peak concentration of 232 mg/dL (12.9 mmol/L) and a late glucose nadir of 29 mg/dL (1.6 mmol/L) [Fig. 1(e)]. Plasma immunoassay insulin was concomitantly >6945 pmol/L (C-peptide not measured). Glycemic lability was confirmed by CGMS [Fig. 1(f)].

Gross hyperinsulinemia was confirmed using MS (Table 2). Insulin measurement by immunoassay underestimated total insulin in neat plasma and was nonlinear to dilution, with very low insulin recovery following PEG precipitation, suggesting IA. GFC showed predominantly HMW insulin immunoreactivity, confirming IAS [Fig. 3(d)]. IAs were strongly positive by ELISA but equivocal by RIA, the former result consistent with GFC findings of a high insulin-binding capacity. Unlike the low levels of RIA binding with protein A immunoprecipitation (Table 2), high levels were demonstrable with protein G that could be explained by insulin binding due to IgG3. Competitive insulin-binding studies (Fig. 2) (analyzed in neat serum) suggested a micromolar dissociation constant.

Diazoxide (50 mg thrice daily) reduced the frequency and severity of hypoglycemia, but after 6 months, lanreotide 60 mg subcutaneously was added monthly as hypoglycemia continued to compromise quality of life. Lanreotide controlled hypoglycemia but caused gastrointestinal side effects, leading to its withdrawal. Acarbose was not tolerated. Diazoxide was continued at an increased dose (100 mg thrice daily) for 3 years with concomitant diuretics to manage edema. HbA1c on diazoxide remained around 55 mmol/mol (NR 20 to 42). Immunomodulatory therapy was declined but remains under consideration.

Patient 5 presented with recurrent falls associated with cognitive decline. Borderline low CBG concentrations at 50 mg/dL (2.8 mmol/L), as well as concentrations as high as 248 mg/dL (13.8 mmol/L) consistent with diabetes mellitus, were noted during admission, but no glycopenic symptoms were apparent. Plasma immunoassay insulin concentration, at a time when blood glucose concentration was 37 mg/dL (2.1 mmol/L), was 1024 pmol/L with a concomitant C-peptide of 679 pmol/L and insulin to C-peptide molar ratio of 1.51 (NR 0.03 to 0.25). Gross hyperinsulinemia was confirmed using MS (Table 2). Insulin measurement by immunoassay underestimated total insulin in neat plasma and was nonlinear to dilution, with very low insulin recovery following PEG precipitation, suggesting IA. GFC showed HMW insulin immunoreactivity, confirming IAS. IAs were positive by ELISA and RIA, and competitive insulin-binding studies (Fig. 2) (analyzed at 10-fold serum dilution) suggested a submicromolar dissociation constant. Further investigation and treatment were declined, and the patient was discharged to residential care with a CBG meter and advice to avoid fasting. Four months later, she was admitted to the hospital with reduced consciousness and a CBG reading of 23 mg/dL (1.3 mmol/L). Blood glucose normalized with intravenous glucose. Prednisolone 10 mg daily was commenced and the patient was discharged with advice for regular blood glucose monitoring, and glucose gel was provided. She has since died.

Patient 6 presented with two episodes of loss of consciousness due to hypoglycemia. On both occasions, low CBG was detected, and he was admitted to the hospital for emergency treatment. He had no family history of diabetes or hypoglycemia. Two 72-hour fasts failed to provoke hypoglycemia, with a glucose nadir during the first fast of 72 mg/dL (4.0 mmol/L). In contrast, prolonged 75-g OGTT produced a glucose nadir of 26 mg/dL (1.4 mmol/L) [Fig. 1(g)] with a corresponding immunoassay insulin of 1285 pmol/L, C-peptide of 1006 pmol/L, and insulin to C-peptide ratio of 1.28 (NR 0.03 to 0.25) at 180 minutes after the glucose load. This led to loss of consciousness, which was rescued with intravenous glucose. IAS was suspected, and prednisolone 60 mg with diazoxide 300 mg daily was commenced. IAs were, however, within reference limits using two RIAs.

Gross hyperinsulinemia was confirmed using MS (Table 2). Insulin measurement using immunoassay underestimated total insulin in neat plasma and was nonlinear to dilution, with low insulin recovery following PEG precipitation, suggesting IA. GFC studies with and without preincubation of plasma with exogenous human insulin showed HMW insulin immunoreactivity consistent with insulin-binding antibodies, confirming the diagnosis of IAS [Fig. 3(e)]. IAs were equivocal by ELISA and negative by RIA, which was inconsistent with GFC findings of a high insulin-binding capacity. To identify the class of the putative IA, immunosubtraction studies were performed using antibody class-specific antibodies conjugated to agarose. Patient 6 plasma was compared with control plasma with insulin-binding IgG and three plasma samples with no evidence of insulin autoimmunity, all matched for insulin concentration. To increase the sensitivity of the method to detect IA, plasma was incubated with synthetic human insulin to drive the binding equilibrium in favor of bound insulin. Plasma insulin recovery was close to 100% in all cases except for those with anti-insulin IgG subtracted for IgG and patient 6 subtracted for IgA. In both cases, recovery fell to around 50% to 60%, indicating the presence of anti-insulin IgA in patient 6. In keeping with this, no increased precipitation of radiolabel was seen using either protein G or protein A, but demonstrably increased precipitation was seen with anti-IgA agarose. The baseline PAS/PGS radioligand binding was too low (analyzed in neat serum) to allow reliable calculation of binding affinity.

Prednisolone was reduced to 40 mg daily, and no further symptomatic hypoglycemia was recorded. Four months following diagnosis, during prednisolone treatment, blood tests confirmed the continued presence of insulin-binding antibodies. CGMS confirmed labile glycemia, with matutinal hyperglycemia and postprandial hyperglycemia [Fig. 1(h)] leading to immunodepletion therapy being considered.

### Quantitative liquid chromatography–MS insulin and C-peptide results

Individual results are shown in Table 2. There was insufficient plasma from patient 2 for analysis. Molar ratios of IAS insulin/C-peptide ranged from 3.7 to 8.4, and for 34 control plasma samples, they ranged from 0.2 to 1.5, with one outlier of 0.02.

## Discussion

IAS has been reported most widely in Japan (1), and despite numerous but scattered reports elsewhere and frequent airing of the diagnostic possibility in forensic investigation of suspected insulin poisoning, there is relatively little awareness of the condition in the Western Hemisphere among endocrinologists. IAS most often presents with hypoglycemia, which may be postprandial, postabsorptive, or fasting. In this series, the presenting symptoms ranged from daytime loss of consciousness to modest symptoms only after overnight fasting. Patients 1, 4, and 6 displayed reactive hypoglycemia on dynamic testing, whereas in patients 2 and 3, hypoglycemia was provoked by fasting. Prolonged fasting of patients 1, 4, and 6 did not result in hypoglycemia using thresholds aimed at excluding insulinoma, as in some published cases of IAS (16, 26, 27). Four of six patients underwent imaging using modalities including MRI, endoscopic ultrasonography, and positron emission tomography/single-photon emission computerized tomography before IAS was diagnosed. Suggestive biochemical evidence for IAS existed in each case, and some imaging may have been avoided with earlier access to definitive testing.

In this series, the first clue to IAS came from high insulin concentrations and insulin/C-peptide molar ratios in samples drawn during hypoglycemia. Immunoassay results were shown to be nonlinear to dilution at presentation (linearity improving following plasma exchange) and to underestimate MS-detected insulin in neat plasma, consistent with assay interference due to the IA competing with the immunoassay antibodies for insulin-binding sites (6). Consistent with previous observations (28–31), immunoassay C-peptide concentrations, in the five patients in whom they were measured, were reported at hundreds to thousands of picomoles per liter, concurrent with hypoglycemia. Immunoassay C-peptide concentrations in patients 1, 3, 4, and 6 conversely overestimated MS C-peptide more than may be expected from assay bias alone (32), possibly due to cross-reacting insulin precursors not detected by the MS method. As MS methods are not susceptible to antibody interference, they are more likely to return a correct value for total insulin concentration in IAS and thus increased confidence in the diagnosis.

IAs are a *sine qua non* of IAS (33), but assay sensitivity and specificity in the diagnosis of IAS have not been established. Indeed, IAs were first described in patients receiving exogenous insulin (34, 35) with such frequency that in early literature, the presence of such antibodies in hypoglycemic ostensibly insulin-naive patients was regarded as nearly diagnostic of surreptitious insulin administration (36). They are now well established in the repertoire of autoantibodies used to identify type 1 diabetes (37) and to stratify nondiabetic people according to the risk of autoimmune diabetes (38, 39). They may also be detected in healthy blood donors or patients with unrelated autoimmune disorders (40–42). Different diagnostic laboratories use different methods; these are nonstandardized, and assay concordance remains poor (5, 43) despite longstanding attempts at harmonization (44). In all patients, recovery of immunoreactive insulin after PEG precipitation was low and GFC confirmed HMW insulin-containing complexes, but not all patients had elevated IA on initial testing. In this study, ELISA and RIA moreover produced different rankings of the magnitude of the results, possibly due to differential effects of high endogenous insulin concentrations. Antibody characteristics will also contribute to assay variability: for patient 4, the ELISA/RIA discrepancy may be attributable to underrepresentation of IgG3 in immunoglobulins captured by protein A prior to RIA. More strikingly, in patient 6, equivocal or negative antibody levels were determined using four different IA assays, despite convincing GFC evidence of insulin-antibody complexes. Anti-insulin IgA was ultimately demonstrated by immunosubtraction, explaining the discrepancy. Only ∼70% of IgA is removed using PEG precipitation (in-house data), explaining the relatively modest suppression of recovery after PEG precipitation in this case and raising the possibility that PEG precipitation may offer false reassurance in the presence of IgA IA. The use of alternative immunoprecipitation methods may increase the sensitivity of these tests but offer diminishing returns and increase complexity and cost. For example, further studies using anti-IgA showed patient 5 also to possess notable IgA IA binding of insulin. Unfortunately, there is no failsafe method for immunosubtraction of immunoglobulin subclasses. It is tempting to speculate that patients 2 and 3 exhibited hypoglycemia principally during fasting due to the high affinity and very high capacity of their IA, but antibody capacity and affinity did not appear to correlate with physiological abnormality across the whole group studied.

Hypoglycemia in IAS has been reported to resolve spontaneously in most patients within 3 months (1). The severe hypoglycemia seen in this series, sustained over months or years, allied to other reports, demonstrates that this is not always true, however. As IAS is antibody mediated, targeting of pathogenic antibodies is rational. In keeping with this, diazoxide, which targets insulin secretion, showed modest or no benefit. Four patients in this series to date were treated with immunomodulatory therapies. Patient 1 was treated with glucocorticoids alone over >4 months, but intermittent hypoglycemia persisted and so therapy with rituximab was used. Patient 6 also failed to experience improvement of glycemic lability, and immunodepletion therapy is being considered. Patients 2 and 3 were both initially treated with glucocorticoids and MMF, but although patient 2 appears to have gone into remission relatively quickly, patient 3 continued to experience severe hypoglycemia, despite high-dose glucocorticoids (which caused severe side effects). Ultimately, it was necessary to combine plasma exchange with rituximab therapy. Collectively, this demonstrates that therapeutic responses are variable.

In summary, IAS should be considered in cases of spontaneous hypoglycemia with a high insulin/C-peptide molar ratio. Measurement of IA is an appropriate screening step, but although the IA assays used in this series detected antibodies in five patients, they were equivocal or negative in patient 6, illustrating that IA results are assay dependent (5). Moreover, detection of IA alone is not specific for actionable antibodies (6), meaning that further measures to confirm plasma insulin-antibody complexes are required for diagnosis. MS-based methods promise to increase diagnostic confidence as they are unaffected by antibody-based assay interference. Immunodepletion is warranted in severely affected patients. Our series demonstrates that therapeutic responses vary, and so a customized and flexible approach to depleting pathogenic antibodies is required. More standardized approaches to IAS diagnosis will facilitate the systematic therapeutic studies required.

## Abbreviations:

CBG: capillary blood glucose
CGMS: continuous glucose monitoring system
GFC: gel filtration chromatography
HMW: high molecular weight
IA: anti–insulin antibody
IAS: insulin autoimmune syndrome
MMF: mycophenolate mofetil
MS: mass spectrometry
NR: normal range
OGTT: oral glucose tolerance test
PAS: protein A Sepharose
PEG: polyethylene glycol
PGS: protein G Sepharose
